# Assessment of Various Colors Combined with Insecticides in Devising Ovitraps as Attracting and Killing Tools for Mosquitoes

**DOI:** 10.3390/insects14010025

**Published:** 2022-12-26

**Authors:** Adam Khan, Misbah Ullah, Gul Zamin Khan, Nazeer Ahmed, Ashwag Shami, Rania Ali El Hadi Mohamed, Fahd Mohammed Abd Al Galil, Muhammad Salman

**Affiliations:** 1 Department of Entomology, Faculty of Crop Protection Sciences, The University of Agriculture Peshawar, Peshawar 25130, Khyber Pakhtunkhwa, Pakistan; adam_khan345@outlook.com (A.K.); misbah_ullah2003@outlook.com (M.U.); 2 Medical Entomology Laboratory, Nuclear Institute for Food and Agriculture (NIFA), Peshawar 25130, Khyber Pakhtunkhwa, Pakistan; gz_k213@outlook.com (G.Z.K.); salman_khan329@outlook.com (M.S.); 3 Department of Agriculture, University of Swabi, Anbar 23561, Khyber Pakhtunkhwa, Pakistan; 4 Department of Biology, College of Science, Princess Nourah bint Abdulrahman University, P.O. Box 84428, Riyadh 11671, Saudi Arabia; ayshami@pnu.edu.sa (A.S.); raniaelhadi@yahoo.com (R.A.E.H.M.); 5 Department of Biology, College of Science, University of Bisha, P.O. Box 551, Bisha 61922, Saudi Arabia; fahd.bamu78@gmail.com

**Keywords:** mosquito, ovitrap, insecticides, food material, management

## Abstract

**Simple Summary:**

The Aedes mosquito species are highly adapted to human dwellings where they develop in a large variety of human-made containers, and adults feed on human blood, resulting in the transmission of deadly disease agents. Therefore, the development of attracting and killing tools in the form of traps is thus required In the current studies, our primary focus was on developing low-cost monitoring tools to be exploited for the ultimate suppression & control of dengue vector mosquitoes. The attractive baited traps have shown promising results in laboratory settings and have the potential to significantly reduce the Aedes mosquito populations in the pilot field as well. Monitoring trials in the selected sites were deployed for the population fluctuation of dengue vectors and were compared with the surrounding buffer neighborhood with no traps. Entomological survey data from the study was utilized for recording the reduction in egg-laying mosquitoes in the neighborhood with traps. These traps were designed to reduce the number of mosquitoes through two main actions: (1) Attraction: Aedes species were attracted by the combination of odors emitted by baits in the water inside the trap and the black color of the trap; (2) Adulticide: the inside walls of the trap were lined with insecticide deltamethrin that kills resting adults.

**Abstract:**

Dengue virus, transmitted by *Aedes aegypti* mosquitoes, is the most important emerging viral disease, infecting more than 50 million people annually. Currently used sticky traps are useful tools for monitoring and controlling *Ae. aegypti*. Therefore, this study was conducted to evaluate the attraction of *Ae. aegypti* mosquitoes using various colors, materials and insecticides. The laboratory and field assessed the four different colors of ovitraps (blue, green, black and transparent). Among the tested ovitraps, the black ovitraps showed the highest number of eggs (348.8) in the laboratory and maximum eggs (80.0) in field trials. In addition, six different materials (casein, urea, yeast, fish meal, chicken meal and water) were also used to evaluate mosquito’s attraction. In our results, the highest number of eggs were collected with fish meal having 0.5% concentration in both laboratory (195.17) and the field (100.7). In laboratory trials, the Deltamethrin treated ovitraps (treated with Deltamethrin) significantly trapped and killed the highest percent of female *Ae. aegypti* (91.5%) compared to untreated (not-treated with Deltamethrin) ovitraps (3.3%). In field trials, the lethality was determined by installing 10 lethal ovitraps in one block and 10 untreated ovitraps in another block. The results indicate a significant reduction in eggs collected from the treated block (727 eggs) as compared to the untreated block (1865 eggs). The data also reveal that the ovitrap positive index (50) and egg density index (24.3) were also low in treated areas than in untreated areas, 83.3 and 37.3, respectively. It is concluded that the lethal ovitraps significantly reduced the *Ae. aegypti* population and thus could be considered an integral part of the integrated vector management (IVM) program.

## 1. Introduction

Dengue fever is a disease transmitted by *Aedes* mosquitoes and spread in the tropical and subtropical areas of the earth [1,2]. In 2005, International Health Regulation urges the world community that dengue fever may create an international public health emergency [3]. Recently, it was estimated that about 97 million people were at high risk to be infected per year [2,4]. Dengue is a widespread disease in many countries [5] Numerous mosquito control approaches have been engaged to decrease *Ae. aegypti* population and restrain dengue transmission with different effects [6]. However, intensive elimination of *Ae. aegypti* mosquitoes and various mosquito control approaches greatly affect non-target organisms. In the Mosquito Magnet Pro^®^ (Lititz, PA, United States) field trials, 43,464 insects were trapped over three months, and 395 mosquitoes were captured (target organism). Of these, 43,464, half, and 21,572 were non-biting insects and were considered beneficial [7].

Several mosquito control programs have studied the replacement of broad-scale insecticide applications to ensure less damage to non-target species and decrease the effect on the environment [6], with possible unintended effects on the broader food chain [8]. The utilization of lethal ovitraps has a significant impact on the oviposition activity of *Ae. aegypti*, preventing the vector control staff, the public and non-target species from chemical contact [2,9,10,11]. Lethal ovitraps (Los) have fewer adverse effects on the surrounding environment and are considered eco-friendly and viable [11]. Gravid female *Ae. aegypti* was highly attractive to bifenthrin-treated LOs [10] and decreased field population in Cairns [12]. The combined strategies successfully control *Ae. aegypti* population in Australia since 2004 were the use of lethal ovitraps, larval control, synthetic pyrethroids such as lambda-cyhalothrin and deltamethrin and the use of synthetic pyrethroids [12,13]. Though, the DENV-3 strain that caused an epidemic in 2008–2009 has fast transmission cycles and surpasses control strategies [11,14]. LOs containing glue strips also reduced female *Ae. aegypti* density; however, the effect on non-target species was not studied [15].

Lethal Ovitrap means “lure and kills” trap. The attractant inside ovitraps attracts target insects, and treated ovistrips (a form of ovitrap) kill them when interacting with them, reducing the population of target insects when installed on a large scale [16,17,18]. The ovitraps containing hay infusions are more attractive and lethal if insecticides added to ovistrips were placed in these ovitraps [19]. The *Bacillus thuringiensis* and buprofezil insecticides were used in traps that controlled larvae and adults [20]. Lethal ovitraps are very operative in managing *Aedes* by entirely preventing the larvae and decreasing egg density. The use of lethal ovitraps has no harmful effect on the environment and less installation cost. It is an easy-to-use and powerful tool against *Aedes* [16,17]. This study aims to develop cost-effective, attractive, environmentally friendly traps that are lethal for different mosquito species to reduce the threat of dengue vectors and the deadly disease.

## 2. Materials and Methods

The study was conducted at the Medical Entomology Laboratory, Nuclear Institute of Food and Agriculture (NIFA), Peshawar. Mosquitoes population were reared according to the procedure described by Khan et al. [21].

### 2.1. Preparation of Ovitraps

Plastic bottles were bought from a local scrap shop and cut into two halves (12 cm in height; 8.5 cm in diameter) in such a way to accommodate 500 mL water/solution and were washed with detergents and sun-dried before use. The cut bottles were covered with different colored plastic bags, i.e., black, blue, green, and transparent. The entire internal wall of the ovitraps was covered with filter paper [22].

### 2.2. Laboratory Procedure for Color and Different Attractive Materials Experiments

About 180–200 adult *Ae. aegypti* mosquitoes (1:1) were released in each cage (64 × 64 × 64 cm). The male *Ae. aegypti* mosquitoes were fed on 10% sugar solution, Albino mice were set in the cages for only 20–30 min for blood feeding by female mosquitoes on daily basis. The practice was repeated for 3 days/week. The ovitraps were half-filled with an attractive solution/water and then placed in cages. The Ovistrips were collected after a week, each ovistrip was properly labeled, and eggs were counted on each ovistrip with the help of a binocular dissecting microscope [23]. Dead mosquitoes were replaced by placing cups with pupae in the cage. All stages of *Ae. aegypti* were present in the laboratory. Laboratory temperature was kept 25 ± 10 °C, 55 ± 15% relative humidity (RH), and photoperiod of 10:14 h [19].

#### 2.2.1. Experiment 1

##### Attraction of Gravid Females to Various Colored Ovitraps in Laboratory

Ovitraps covered with different colored plastic bags (black, blue, green and transparent) will be half-filled up with water and placed in the cage to evaluate the most attractive color to which gravid *Ae. aegypti* females were attracted. 

##### Coloured Ovitraps in the Field for Attracting *Ae. aegypti* Females

Coloured ovitraps, as mentioned above, were placed in a shady area (under a tree or any other shelter) where evaporation from ovitraps was minimum. The ovitraps were installed between 09 am–12 pm. The ovitraps were replaced upon any damaged. The distance among the ovitraps was about 3 m from each other. Ovitraps were active up to 4 days and therefore, the traps were replenished after weekly with the required attractants plus water.

#### 2.2.2. Experiment 2

##### Oviposition Preference of Gravid Females to Different Attractive Materials in the Laboratory

In the ovitraps, different attractive materials (chicken meal, fish meal, urea, casein, and yeast) of various concentrations of 0.5%, 1% and 2% (0.5 gm/100 mL, 1 mg/100 mL and 2 gm/100 mL) were added to evaluate the most optimum concentration of material. At the same time, tap water was used as a control.

##### Ovitraps with Attractive Materials Installed in the Field

In the field, ovitraps with different attractive materials were placed in a shady place (under a tree or any shelter) where evaporation from traps was minimum. The distance among ovitraps was about 3 m from each other. If ovitraps were missing or damaged, then the missing ovitraps were replaced by new ovitraps. 

#### 2.2.3. Experiment 3

##### Preparation of Lethal Ovitraps to Control *Ae. aegypti* in Laboratory

The above experiments used a combination of the most attractive color with attractive material in the ovitrap. The ovitraps were lethal by treating their ovistrip with insecticide (deltamethrin 10 mL/L). In this experiment, the cages were cleaned, and 100 pupae of *Ae. aegypti* were placed in each cage. After 4–6 days of the adult mosquito’s emergence, the mosquitoes were given 10% sugar solution. Albino mice were set in the cages for only 20–30 min for blood feeding by female mosquitoes on daily basis. The practice was repeated for 3 days/week. One cage received deltamethrin 10 mL/L treated ovistrip and the other received untreated ovistrip The data was collected after 48 h. Dead mosquitoes (mosquitoes that do not move) were collected, and living mosquitoes were aspirated.

##### Lethal Ovitraps Used in the Field

Before the lethal ovitraps are installed in the field, a six-day survey was conducted in Tarnab-Peshawar, to collect adult mosquitoes with the help of a mechanical aspirator [24]. The collection was done early morning hours (08 am–10 am) for 20 min [25]. The collected mosquitoes were carried to the laboratory for species identification [24] via ID key [25]. The lethal ovitraps were installed in the field in a shady area. Two places were selected for the installation of treated and untreated ovitraps. In one place (equal to 10 marlas = 2722.5 square feet), ten lethal ovitraps were installed, and in another place of the same size, ten untreated ovitraps were placed. After the installment of treated and untreated ovitraps, ovistrips were collected for egg collection every week for up to six weeks. Every 3rd or 4th-day ovitraps were filled with water up to the appropriate level, about half of the filter paper.

#### 2.2.4. Statistical Analysis

Data were analyzed using Completely Randomized Design (CRD) one/two factors. The field data of lethal ovitraps were estimated through egg density index (EDI) and ovitraps positive index (OPI) [25]. Ovitrap positive index was estimated as the percent LOs positive for eggs from the total number of ovitraps inspected.

OPI = No. of lethal ovitraps positive for eggs/Total no. of lethal ovitraps inspected × 100

The efficiency of lethal ovitraps in eggs collection (EDI) was calculated as the average number of eggs laid per positive lethal ovistrip 

EDI = Total no. of eggs on ovistrip/no. of positive ovitraps

The Levene test about the homogeneity of variances prior to ANOVA was conducted. As data were not transformed, therefore, homogeneity-of-variance assumptions were not violated according to the Levene test. Mean values and Standard Deviation (SD) were calculated from included replications. The LSD test of multiple comparisons separated the calculated attraction/killing means up to one/two ways of ANOVA.

## 3. Results

### 3.1. The Ovipositional Preference of Females to Various Colored Ovitraps in the Laboratory

**Figure 1:**
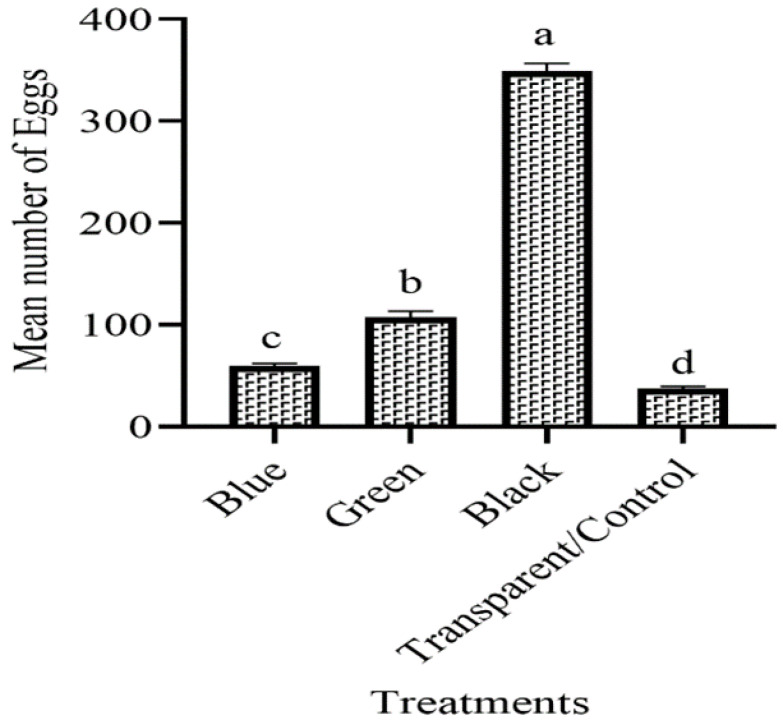
The mean number of eggs of *Aedes aegypti* to various color ovitraps in the laboratory. These letter shows significant difference among treatments.

Female Ovipostional preference showed a significant difference (F = 75.4, df = 3, *p* = 0.001) to various colored ovitraps. The black ovitraps were the most attractive to gravid females after six trials in the laboratory. The mean number of eggs (59.50, 107.50, 348.83, 37.16) were collected from their respective colored ovitraps blue, green, black, and transparent (control). Black ovitraps showed the highest number of eggs (348.83), followed by green (107.50), blue (59.50), and transparent ovitrap (37.16), which means gravid females favored laying their eggs in black ovitrap as compared to others (Figure 1).

### 3.2. The Attraction of Ae. aegypti to Different Colored Ovitraps in the Field

**Figure 2:**
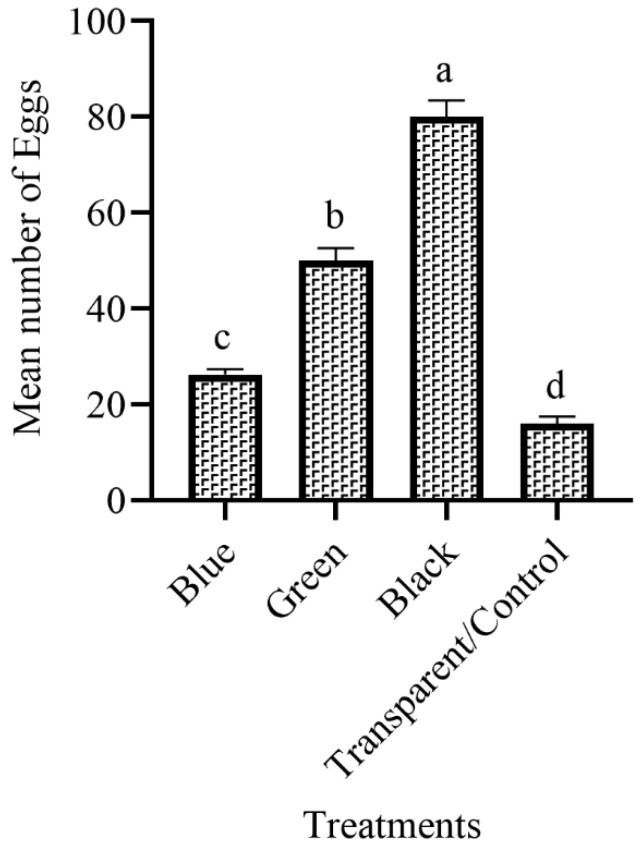
The mean number of eggs of *Ae. aegypti* to various color ovitraps in the field. These letter shows significant difference among treatments.

Figure 2 shows the response of *Ae. aegypti* towards various colored ovitraps in the field. *Ae. aegypti* showed significant (F = 14.3, df = 3, *p* = 0.01) attraction in the field ovitraps. The black ovitraps were prominent and attracted more females than other colored ovitraps. The highest number of eggs was recorded from black ovitraps (80.00), and the lowest was collected from transparent/control (16.00). Overall, the results of field experiments showed that black ovitraps were the most attractive, followed by green, blue and transparent (Figure 2).

### 3.3. Effect of Different Attractive Materials with Various Concentrations on Female Oviposition in Black-Colored Ovitraps in Laboratory

**Figure 3:**
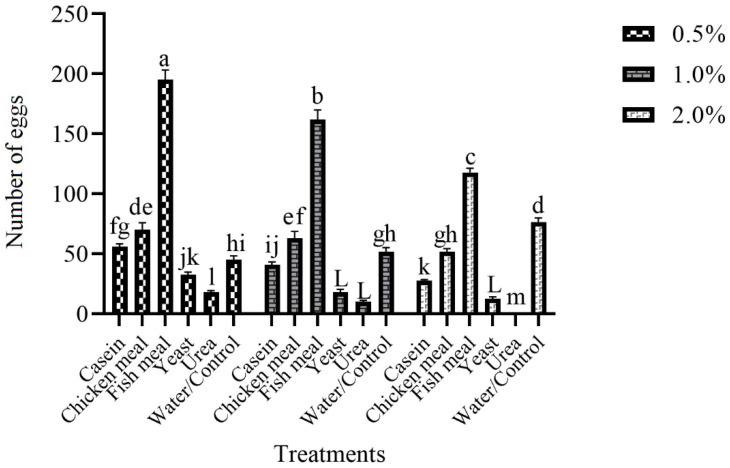
The interaction of attractive materials and concentration in black ovitraps in the laboratory. These letter shows significant difference among treatments.

Figure 3 shows the attraction of different materials to *Ae. aegypti* in the laboratory. Different attractive materials with various concentrations on female oviposition showed a significant difference (F = 6.1, df = 5, *p* =  0.005) in black-colored ovitraps. The highest number of eggs was recorded at 0.5% concentration of fish meal ovitraps (195.17) and the lowest at 2.0% concentration of urea (0.00). At 0.5% concentration, the highest number of eggs was recorded in the fish meal (195.17), followed by a chicken meal (70.00), casein (56.00), water (44.83), and yeast (32.33), and urea (18.00). A similar trend was observed at a 1.0% concentration of various materials. The highest numbers of eggs at 1.0% concentration were observed in the fish meal (161.83) and the lowest in urea (10.00). At 2.0% concentration, fish meal showed the highest number of eggs (117.83), followed by water/control (76.50), chicken meal (51.67), casein (27.33), and yeast (12.67) and urea (0.00). At 1.0% and 2.0%, concentration water collected more eggs than urea, casein and yeast. Overall, results showed that fish meal showed the highest number of eggs at every concentration compared to other materials. Results also revealed that an increase in the concentration of materials decreases the attractiveness of *Ae. aegypti.*

### 3.4. Ovipositional Response of Gravid Females of Ae. aegypti to Various Attractive Materials with a Different Concentration in the Field

**Figure 4:**
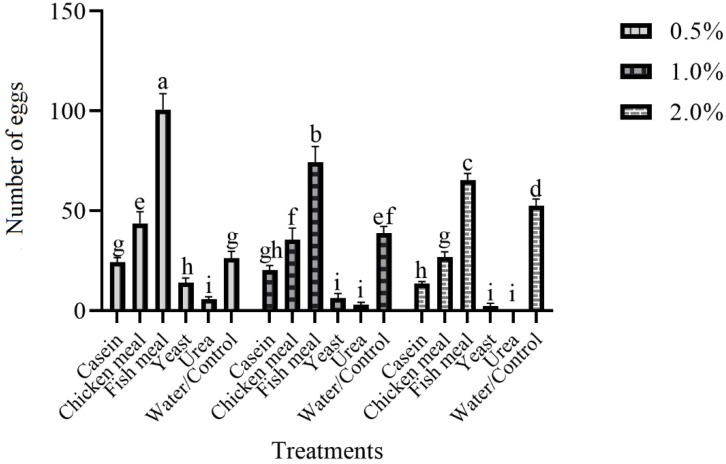
Results of various attractive materials with different concentrations on female oviposition in black-colored ovitraps in laboratory. These letter shows significant difference among treatments.

Figure 4 revealed the attraction of gravid females towards different materials with various concentrations in the field was significantly different (F = 41.17, df = 5, *p* =  0.001). At 0.5% concentration of fish meal (100.75) was more attractive than other materials used in ovitraps, followed by a chicken meal (43.75), water (26.25), casein (24.25), yeast (14.00), and urea (5.75). At 0.5% concentration, all materials were significantly different from each other except water and casein. At 1.0% concentration, the highest number of eggs were found in fish meal ovitraps (74.25), and the lowest was found in urea (3.0). Additionally, at 2.0% concentration again, the highest number of eggs were found in fish meal ovitraps (65.25), and the lowest was found in urea (0.00). As concentration increases, attraction toward materials decreases.

### 3.5. Lethality of Ovitraps by Treating Them with Insecticides in the Laboratory

**Figure 5:**
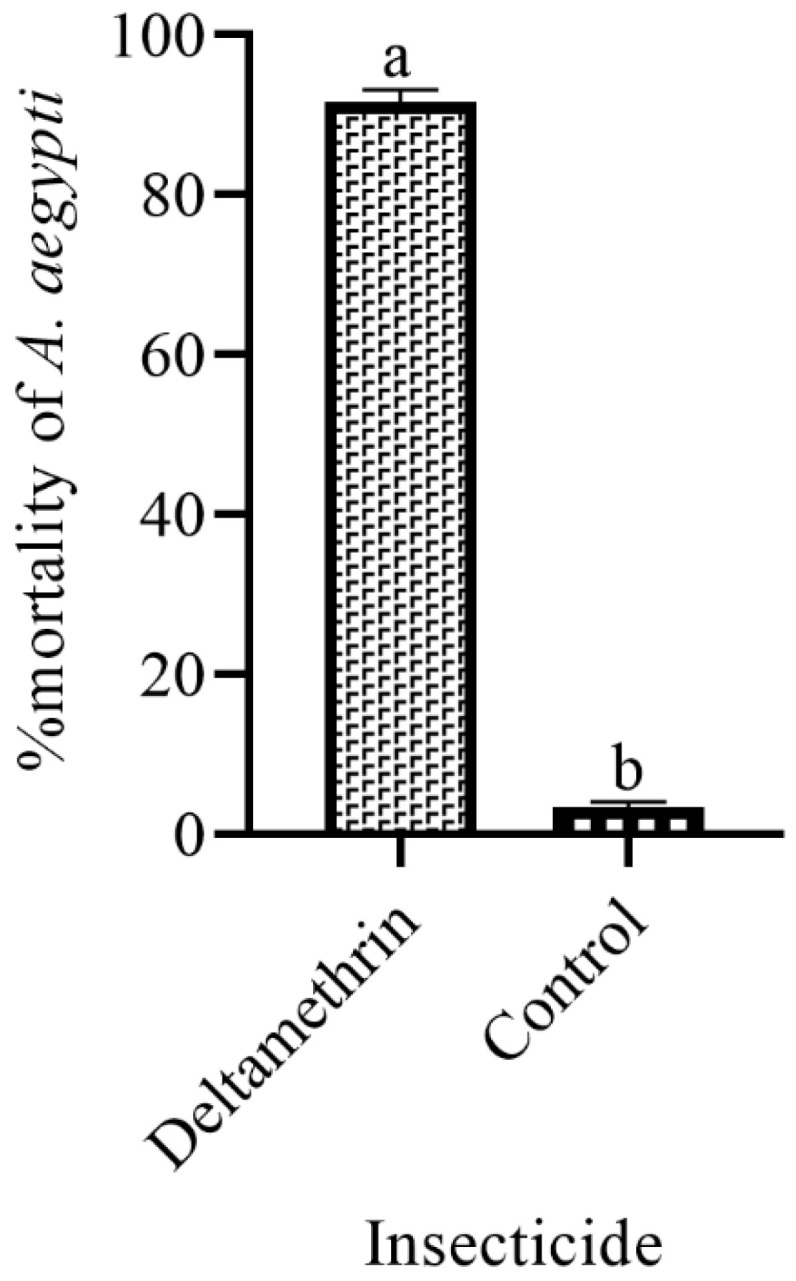
% mortality of females *Ae. aegypti* to different insecticides. These letter shows significant difference among treatments.

Figure 5 showed the mortality of gravid females inside the laboratory ovitraps lethality by treating them with insecticides showed a significant difference (F = 23.60, df = 1, *p* = 0.001). Results revealed that deltamethrin-treated ovitraps resulted in 91.50% mortality during six trials. The ovitraps showed toxic effects on the females after contacting lethal ovistrips to lay their eggs. The results confirmed that *Ae. aegypti* adults could be controlled through lethal ovitraps (Figure 5).

### 3.6. The Effects of Lethal Ovitraps in the Field

Table 1 revealed the activity of *Ae. aegypti* mosquitoes for 6 weeks in both treated and untreated ovitraps blocks shows a significantly different (F = 12.9, df = 1, *p* = 0.001) in the laboratory. A total of 2592 eggs were collected from both blocks. A total of 727 eggs were collected From the LOs block, while 1865 eggs were collected from the control block. The ovitrap positive index (OPI) was also low (50) in the treated block as compared to the untreated block (83.3). The egg density index (EDI) was low (24.2) in the treated ovitraps area as compared to the untreated ovitraps (37.3) area.

### 3.7. The Population of Adults Ae. aegypti before Installation of Lethal Ovitraps

**Figure 6:**
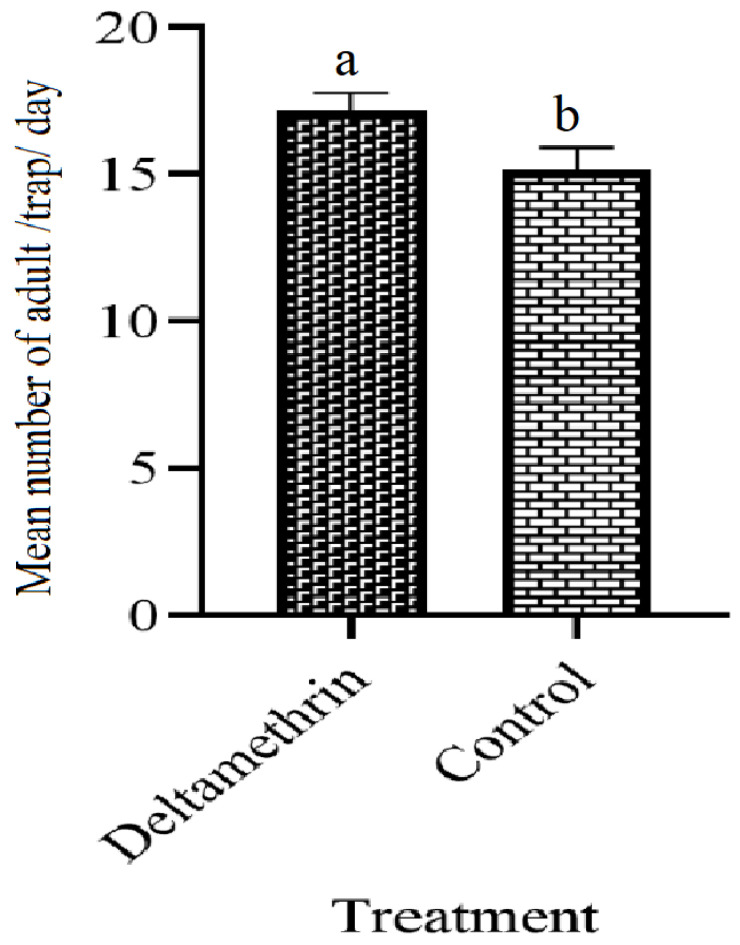
Adults collection from treated and untreated areas before installation of lethal ovitraps. These letter shows significant difference among treatments.

Figure 6 revealed the population of adults *Ae. aegypti* before the installation of lethal ovitraps. The population collected from both areas was significantly different (F = 24.0, df = 1, *p* = 0.001).

### 3.8. Adult Ae. aegypti Density after the Collection of Lethal Ovitraps

**Figure 7:**
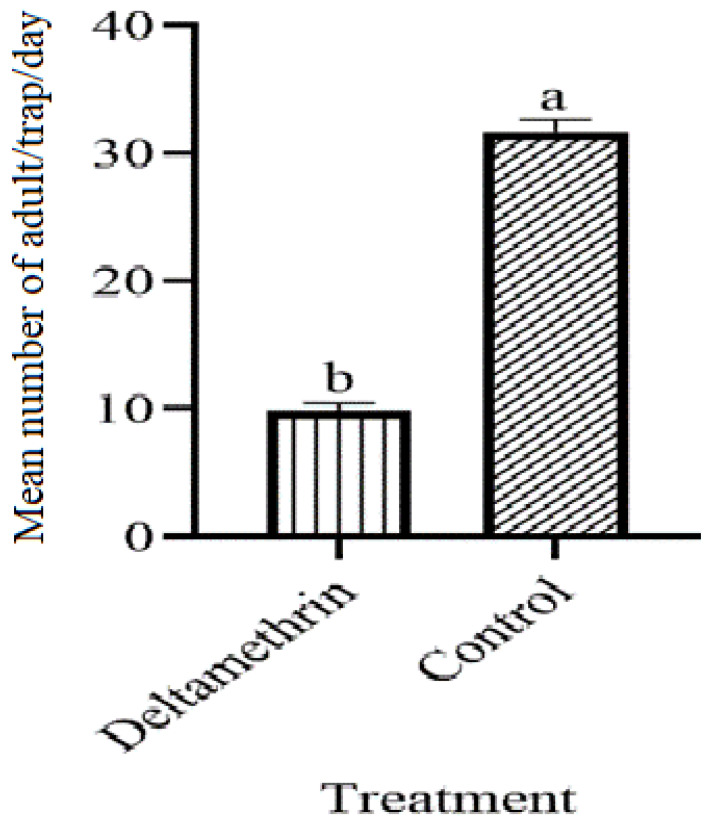
Adults’ collection of *A. aegypti* after lethal ovitraps installation. These letter shows significant difference among treatments.

Figure 7 shows the reduction of the population of adults density after lethal ovitraps were collected. The deltamethrin-treated ovitraps had a significant lethal effect on adult density. These showed significant difference (F = 6.2, df = 1, *p* = 0.001). Adults aspirated from the treated area (9.83) were compared with the control area (31.66), in which females were more active in the control block than in the treatment block.

## 4. Discussion

The choice of place for oviposition is very important in the mosquito’s life cycle. The *Ae. aegypti* nowadays mostly breed in the container placed inside homes. Understanding the oviposition behavior of *Ae. aegypti* may help in dengue surveillance [26]. Size, light intensity, depth, turbidity, PH, the container’s color, and dissolved oxygen were the causes that changed the oviposition behavior of gravid females [27]. In mosquito life, vision plays an important role, including food, oviposition places, mates, the position of hosts, and sleeping places. In our current study, four different colored ovitraps were compared for attracting gravid females in which black color ovitraps showed more mean number of eggs as compared to green, blue and transparent both in the laboratory and in the field. Our results are reliable to the findings of Kumawat et al. [28], who used different color ovitraps for attracting gravid females in which black color was dominant. Marin et al. [29] also found that black color ovitraps were preferred by gravid females as compared to red, green, blue, and orange. Gunathilaka et al. [23] also reported that *Aedes* laid more eggs in black-colored ovitraps and the least number of eggs in white ovitraps. These results showed the fast attraction of *Aedes* females to black-colored ovitraps. Our results also revealed the importance of color while using ovitraps for attracting gravid *Aedes* female mosquitoes.

The oviposition site chosen by the *Aede*s female plays an important role in mosquito dispersal in an area. The checking and surveillance of dengue vectors in dengue hit areas is a huge risk. In the current study, different materials (urea, casein, fish meal, chicken meal, yeast, and water) were used in black-colored ovitraps with various concentrations to attract gravid females to ovitraps. The results revealed that fish meal with 0.5% fish meal showed more eggs than other materials, and the least attraction was found in urea. The results also showed that an increase in the concentration of materials decreases the attraction of the materials. Earlier, researchers studied various materials for attracting gravid females for surveillance. Marin et al. [28] used different plant leaf (*Ocimum tenuiflorum, Hevea brasiliensis,* and *Azadirachta indica)* infusions to increase the attraction of ovitraps. The results showed that *A. indica* leaf infusion showed more eggs than other plant infusions. Gopalakrishnan et al. [29] used hay and leaf infusion to increase the attraction of the trap. The 30% concentration of hay infusion of grass *Pennisetum* and rice straw were dominant compared to banana and mango leaf infusion. Harwood et al. [30] studied different infusions (dried mango leaves *Mangifera indica*, grass *Axonopus* spp., chicken feed pellet) to attract females for oviposition. The results showed that chicken pellet feed collected a high number of mosquitoes compared to grass and mango leaves infusion. All types of infusions were better than plain water. The *Ae. aegypti* was more attractive to *P. maximum* infusion in ovitraps and very effective in mosquito surveillance [31]. Ovitraps enhanced with plant infusions attract more gravid females than traps with plain water by releasing volatiles produced through microbial fermentation [32]. Polson et al. [33] showed that ovitraps are easy to use, low cost, and very effective in dengue surveillance. The current study showed that ovitraps containing fish meal with 0.5% concentration collected more eggs.

In our current results, the mortality rate of *Aedes* females was 91.5% when contacted to lethal ovitraps compared to control in the laboratory. The lethal ovitraps contained an attraction of 0.5% fish meal and deltamethrin recommended on ovistrip The results of the field experiments showed that the use of traps could help reduce the *Aedes* population in an area and compete with other breeding sites. Other researchers also use a different type of lethal ovitraps to suppress the *Aedes* mosquitoes. Our results were similar to William et al. [9], who studied bifenthrin-treated ovitrap in the lab and the field. In a small cage experiment, mosquitoes that laid their eggs in treated ovitraps were 92% killed. In the field trials, bifenthrin-treated ovistrips also showed effective results but were less acceptable to gravid females. Perich et al. [11] studied the evaluation of deltamethrin-treated ovitraps in Brazil. The results revealed that treated ovitraps had significant effects on the density of *Aedes*. Sithiprasasna et al. [34] studied the effects of lethal ovitraps on *Aedes* mosquitoes in Thailand. The study revealed that the *Aedes* population could be reduced through lethal ovitraps, but due to other oviposition sites, the desired efficacy of the lethal ovitraps was not obtained. The main purpose of the research was to develop a low-cost, attractive lethal ovitrap for gravid females.

## 5. Conclusions

In integrated vector control approaches, the trap defined in this current work has the advantage over other control strategies due to the distinctive result of the trap in reducing the *Ae. aegypti* population. Other control plans will be required to control the transmission of the disease because this trap alone is not enough tool to deliver personal protection, similar to other ovitraps. Yet, this trap greatly influences mosquito breeding places and could be a beneficial implement in integrated vector management programs to protect nearby houses and other public places. Community-based deployment, proper examination, and a good place will be required for the traps to decrease dengue disease and its vector. Servicing of the trap will be required every 6–8 weeks, and water will be filled to the appropriate level; add an odor tablet and exchange gauze with the control agents. Though with this regular requirement for servicing, the low price of the trap marks it as an encouraging tool to control the dengue vector.

## Figures and Tables

**Table 1:** Effects of Lethal ovitraps in the field.

S. No	Treatments	No. of Los			Positive Los		Total No. of Eggs	Sum of Total %	EDI
		Installed	Collection	Total	(n)	OPI			
1	LO	10	6	60	30	50	727	28.04	24.2
2	Control	10	6	60	50	83.3	1865	71.95	37.3
3	Total	20	12	120	80	66.6	2592	100	32.4

Los: Lethal Ovitraps.

## Data Availability

Most of the data are available in the manuscript.
